# An Artificial Intelligence-Based Non-Invasive Approach for Cardiovascular Disease Risk Stratification in Obstructive Sleep Apnea Patients: A Narrative Review

**DOI:** 10.31083/j.rcm2512463

**Published:** 2024-12-28

**Authors:** Luca Saba, Mahesh Maindarkar, Narendra N. Khanna, Anudeep Puvvula, Gavino Faa, Esma Isenovic, Amer Johri, Mostafa M. Fouda, Ekta Tiwari, Manudeep K. Kalra, Jasjit S. Suri

**Affiliations:** ^1^Department of Radiology, Azienda Ospedaliero Universitaria, 40138 Cagliari, Italy; ^2^School of Bioengineering Sciences and Research, MIT Art, Design and Technology University, 412021 Pune, India; ^3^Department of Cardiology, Indraprastha APOLLO Hospitals, 110001 New Delhi, India; ^4^Department of Radiology, and Pathology, Annu’s Hospitals for Skin and Diabetes, 524101 Nellore, India; ^5^Department of Radiology, and Pathology, Azienda Ospedaliero Universitaria, 09123 Cagliari, Italy; ^6^Now with Department of Medical Sciences and Public Health, University of Cagliari, 09124 Cagliari, Italy; ^7^Department of Radiobiology and Molecular Genetics, National Institute of the Republic of Serbia, University of Belgrade, 192204 Belgrade, Serbia; ^8^Department of Medicine, Division of Cardiology, Queen’s University, Kingston, ON K7L 3N6, Canada; ^9^Department of Electrical and Computer Engineering, Idaho State University, Pocatello, ID 83209, USA; ^10^Cardiology Imaging, Visvesvaraya National Institute of Technology Nagpur, 440010 Nagpur, India; ^11^Department of Radiology, Harvard Medical School, Boston, MA 02115, USA; ^12^University Center for Research & Development, Chandigarh University, 140413 Mohali, India; ^13^Department of CE, Graphics Era Deemed to be University, 248002 Dehradun, India; ^14^Symbiosis Institute of Technology, Nagpur Campus, Symbiosis International (Deemed University), 440008 Pune, India; ^15^Stroke Diagnostic and Monitoring Division, AtheroPoint™️, Roseville, CA 95661, USA

**Keywords:** obstructive sleep apnea, cardiovascular disease, stroke, cardiac autonomic dysfunction, artificial intelligence, recommendations, deep learning, bias, pruning, explainable, cloud

## Abstract

**Background:**

Obstructive sleep apnea (OSA) is a severe condition associated with numerous cardiovascular complications, including heart failure. The complex biological and morphological relationship between OSA and atherosclerotic cardiovascular disease (ASCVD) poses challenges in predicting adverse cardiovascular outcomes. While artificial intelligence (AI) has shown potential for predicting cardiovascular disease (CVD) and stroke risks in other conditions, there is a lack of detailed, bias-free, and compressed AI models for ASCVD and stroke risk stratification in OSA patients. This study aimed to address this gap by proposing three hypotheses: (i) a strong relationship exists between OSA and ASCVD/stroke, (ii) deep learning (DL) can stratify ASCVD/stroke risk in OSA patients using surrogate carotid imaging, and (iii) including OSA risk as a covariate with cardiovascular risk factors can improve CVD risk stratification.

**Methods:**

The study employed the Preferred Reporting Items for Systematic reviews and Meta-analyses (PRISMA) search strategy, yielding 191 studies that link OSA with coronary, carotid, and aortic atherosclerotic vascular diseases. This research investigated the link between OSA and CVD, explored DL solutions for OSA detection, and examined the role of DL in utilizing carotid surrogate biomarkers by saving costs. Lastly, we benchmark our strategy against previous studies.

**Results:**

(i) This study found that CVD and OSA are indirectly or directly related. (ii) DL models demonstrated significant potential in improving OSA detection and proved effective in CVD risk stratification using carotid ultrasound as a biomarker. (iii) Additionally, DL was shown to be useful for CVD risk stratification in OSA patients; (iv) There are important AI attributes such as AI-bias, AI-explainability, AI-pruning, and AI-cloud, which play an important role in CVD risk for OSA patients.

**Conclusions:**

DL provides a powerful tool for CVD risk stratification in OSA patients. These results can promote several recommendations for developing unique, bias-free, and explainable AI algorithms for predicting ASCVD and stroke risks in patients with OSA.

## 1. Introduction

Atherosclerotic cardiovascular disease (ASCVD) accounted for approximately 800,000 deaths in the United States (US) in 2020, representing 36% of total mortality, with 647,000 of these deaths occurring in individuals over the age of 65 [1, 2]. By 2030, the direct medical expenses associated with ASCVD are projected to exceed USD 920 billion [3]. According to the Sleep Apnea Association (SAA), 38,000 Americans die annually due to the combined effects of ASCVD and obstructive sleep apnea (OSA) [4]. Meanwhile, countries such as China, the US, Brazil, and India report the highest prevalence of OSA globally [5]. OSA affects 34% of males and 17% of females but is often underdiagnosed, with only 10% of OSA patients receiving an appropriate diagnosis and treatment [6]. Severe OSA contributes to an increased mortality rate from ASCVD and is associated with intermittent hypoxia, hypercapnia, and sympathetic overactivity [7].

OSA-related strokes occur when the brainstem fails to effectively communicate with the upper airway or lower thoracic muscles, leading to brain activation, intrathoracic pressure shifts, hypoxia, and reoxygenation due to upper airway collapse [8]. These events decrease oxyhemoglobin saturation and cause electroencephalogram (EEG) arousals [2]. During sleep, these cycles initiate pathways that increase the risk of atherosclerosis [9, 10, 11]. Previous studies have utilized polysomnographic data to predict OSA using machine learning (ML) and deep learning (DL) techniques [1, 2]. Traditional signal-processing methods for predicting cardiovascular outcomes in OSA patients have several limitations [12]. These methods often struggle with the complexity and variability of biological signals and may not effectively capture the multifactorial nature of OSA and ASCVD interactions [13]. Furthermore, they can be biased and fail to provide a comprehensive and explainable risk assessment [14, 15].

However, no study has focused on ASCVD risk stratification in OSA patients. Artificial intelligence (AI)-based systems have been employed to analyze heart rate variability (HRV) events, yet several reports indicate a biological link between OSA and ASCVD. Therefore, including OSA risk as a covariate in cardiovascular risk assessments could enhance the accuracy of ASCVD risk stratification [16, 17].

DL leverages convolution, max-pooling, and attention mechanisms (spatial and temporal attention maps) to extract features and characterize OSA–ASCVD relationships in both signal-based and image-based frameworks [18, 19]. Given the complexity of biological non-linear phenomena, developing robust, accurate, real-time DL paradigms is crucial for detecting OSA and stratifying ASCVD risk [20, 21]. Since evaluating ASCVD risk in OSA patients is challenging due to biological and morphological changes, surrogate biomarkers such as carotid artery disease can be used as indicators for coronary artery disease (CAD) [22, 23, 24].

This study proposes a DL approach to detect high-risk OSA and predict ASCVD risk using a carotid window [25, 26, 27]. We also address clinical evaluation and validation challenges, which can introduce bias and overfitting in DL prediction models [28]. With the trend towards miniaturized medical devices, such as edge devices, reducing the size of DL-based training systems is essential [29, 30]. Therefore, we explored pruned or compressed AI models for assessing ASCVD risk in OSA patients [29, 30]. Additionally, we emphasized the importance of understanding AI “black boxes” through explainability paradigms and extended this into a cloud-based framework [31, 32].

This study aimed to review DL systems for the joint detection of OSA and ASCVD risk stratification [33] while ensuring lower bias, higher compression, and clinical explainability within a cloud/telemedicine framework.

## 2. Search Strategy

**Fig. 1.**
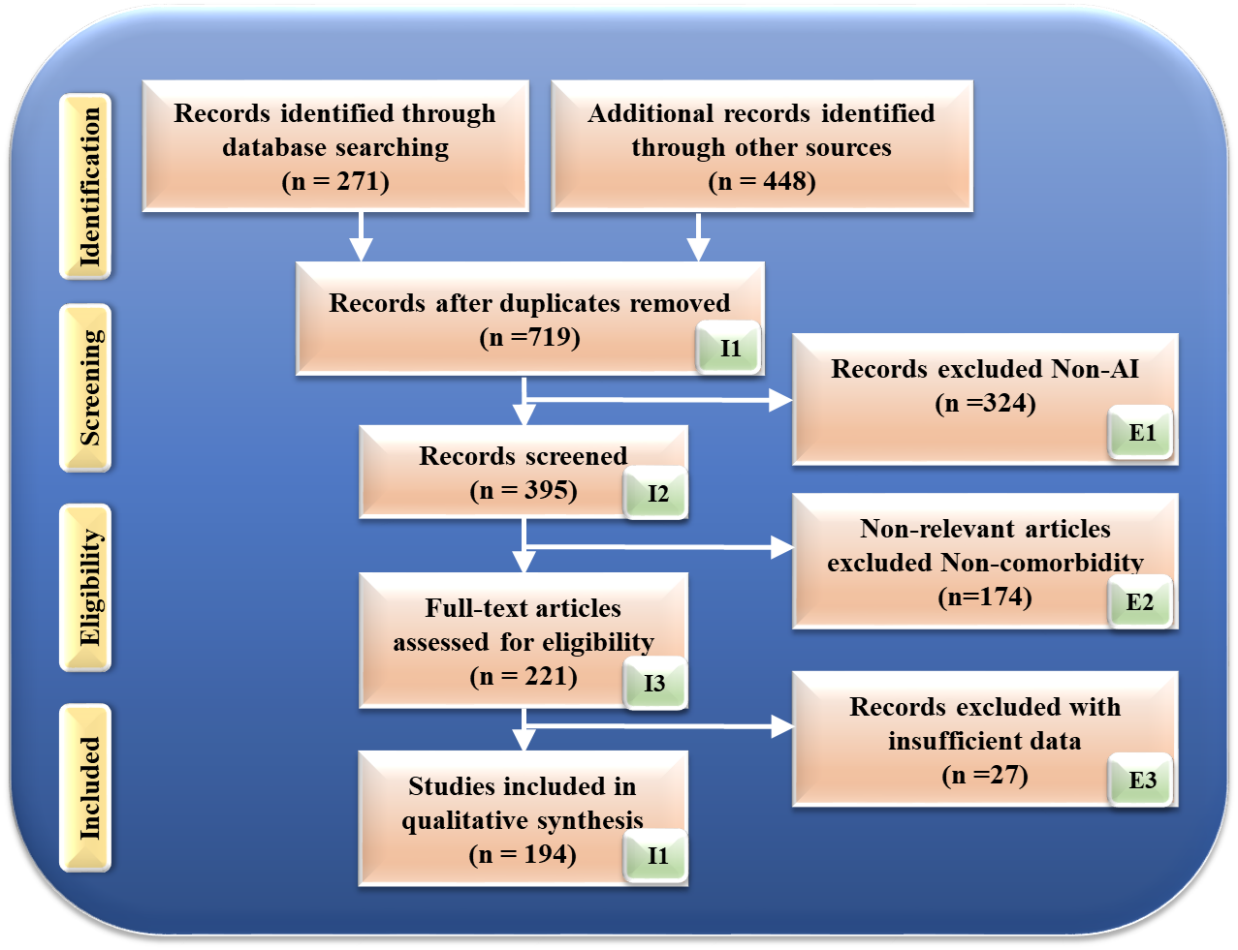
**Search strategy based on the Preferred Reporting Items for Systematic Reviews and Meta-Analyses (PRISMA) model**. AI, artificial intelligence.

Fig. 1 illustrates the search technique based on the Preferred Reporting Items for Systematic reviews and Meta-Analyses (PRISMA) guidelines. This process began with a comprehensive search using specific keywords related to OSA and its associations with cardiovascular disease, stroke, carotid imaging, AI, and deep learning. The search terms included combinations such as “obstructive sleep apnea and cardiovascular disease”, “obstructive sleep apnea and stroke”, “obstructive sleep apnea and carotid imaging”, “obstructive sleep apnea and AI”, “ASCVD atherosclerotic tissue classification and characterization”, “obstructive sleep apnea and deep learning”, “carotid plaque tissue characterization in sleep apnea”, and the conjunction of “obstructive sleep apnea” with databases, including PubMed and Google Scholar, to screen pertinent papers.

Initially, this search yielded 271 records from the specified databases and an additional 448 records from other sources, resulting in 719 records after accounting for quality-specific factors, such as timeliness and relevance. These factors ensured that the included studies were up-to-date and pertinent to the research topic.

The review process involved several stages of filtering. Firstly, studies that were unrelated to the primary focus of the research were excluded, which accounted for 324 studies. Next, irrelevant studies, those that did not directly address the specific research questions or objectives, were also removed, totaling 174 studies. Furthermore, studies with insufficient data, those lacking the necessary information for comprehensive analysis, were excluded, amounting to 51 studies.

After this meticulous exclusion process, 194 research studies were deemed suitable for inclusion in this review. These studies provided the necessary data and relevance to contribute to the systematic review and meta-analysis of OSA and its association with cardiovascular outcomes. This detailed selection process ensured that the final pool of studies was robust, relevant, and sufficient for the intended analysis, offering a comprehensive overview of the existing research on the topic.

## 3. Biological Link Shows that OSA Contributes to Conditions and Mechanisms Involved in ASCVD and Stroke Development

**Fig. 2.**
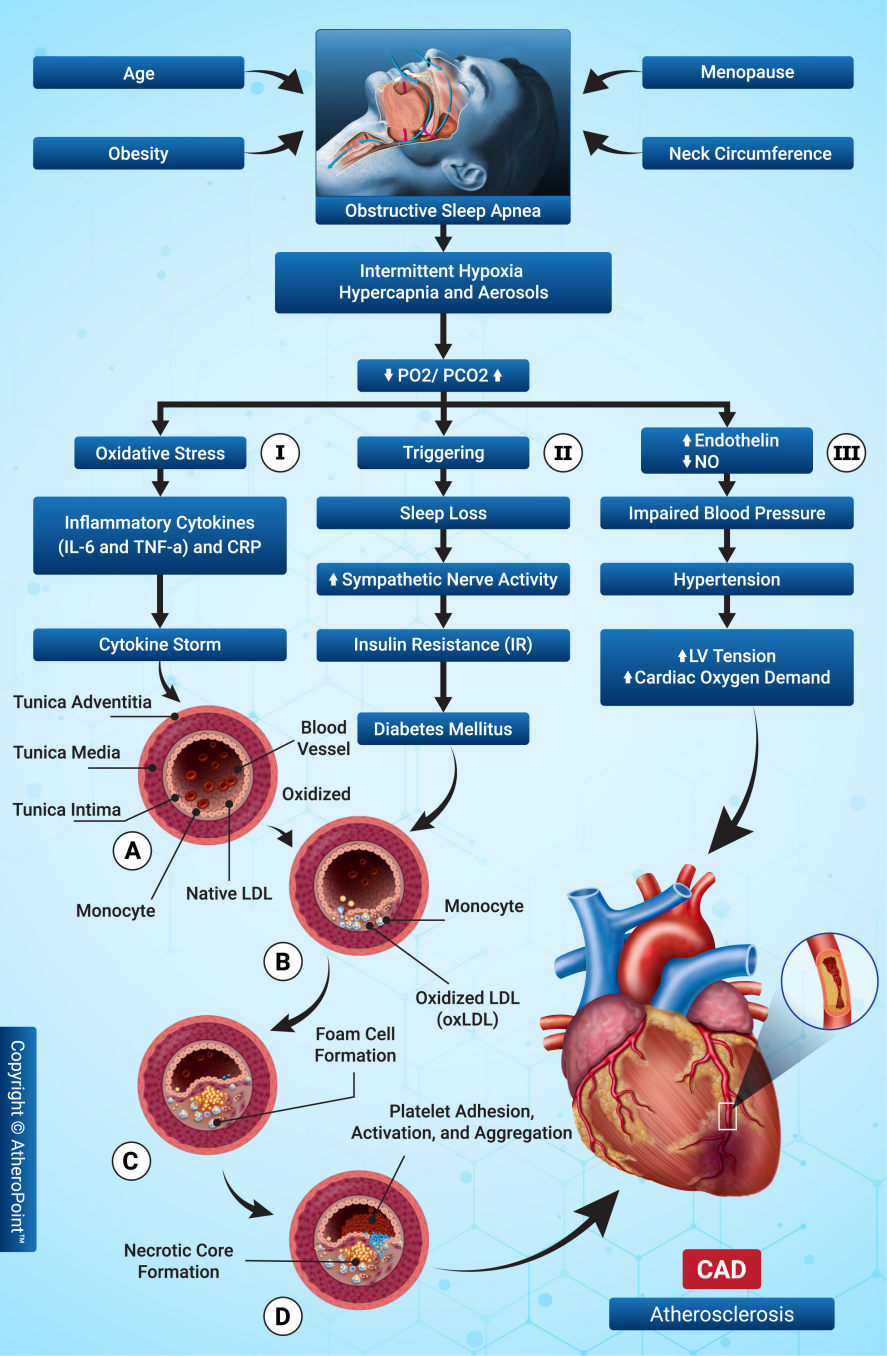
**The biological link between obstructive sleep apnea (OSA) and atherosclerotic cardiovascular disease (ASCVD)**. IL-6, interleukin-6; TNF-α, tumor necrosis factor α; CRP, C-reactive protein, PO2, partial pressure of oxygen; PCO2, partial pressure of carbon dioxide; NO, nitric oxide; LV, left ventricle; LDL, low-density lipoprotein; oxLDL, oxidized low-density lipoprotein; CAD, coronary artery disease.

The pathophysiological link between OSA and the progression of ASCVD remains unknown [34, 35]. However, several pathogenic factors, such as macro and micro-arousals [36], intermittent hypoxia and hypercapnia [37], oxidative stress, inflammation, and vascular dysfunction, have been proposed as intermediate mechanisms linking OSA and ASCVD [38, 39]. Although discussed separately here, these mechanisms are connected and appear sequentially in patients with OSA. The biological mechanism through which OSA leads to ASCVD is depicted in Fig. 2 through three possible routes.

Path I: Hypoxia and hypercapnia, combined with intermittent hypoxia, cause oxidative stress, reactive oxygen species (ROS), inflammatory cytokines, and C-reactive protein (CRP) [40]. Thus, atherosclerotic plaque development in OSA is attributed to the cytokine storm [41], which causes endothelial dysfunction and favors low-density lipoproteins (LDLs) and circulating factors entering the intima of the blood vessels [42]. Moreover, increased inflammatory cytokines and ROS induce the oxidization of LDLs (OxLDL) [43]. Savransky *et al*. [44] reported intermittent hypoxia can cause lipid oxidation and atherosclerotic plaques. Nuclear factor kappa-B (NF-κB)-activated macrophages absorb OxLDL, resulting in foam cells [45, 46]. These foam cells create the necrotic core, forming atherosclerotic plaques that cause ASCVD [47].

Path II: Obesity, OSA, and metabolic dysregulation are linked. Further, OSA, insulin resistance (IR), and ASCVD have the same pathophysiological connection and risk factors. OSA causes behavioral, metabolic, and hormonal disturbances that promote weight gain and IR [48]. Since calories are largely used when resting, these disturbances may activate the neuroendocrine region, promoting hunger and eventual weight gain [49]. OSA increases sympathetic nerve activity (SNA), which may change glucose metabolism, accelerate glycogen breakdown in skeletal muscles, and increase hepatic glucose synthesis. SNA release may raise cholesterol, triglycerides, and IR [50, 51]. IR causes more lipoproteins, which oxidize, increasing OxLDL levels and promoting atherosclerosis and ASCVD.

Path III: Intermittent hypoxia with hypercapnia causes baroreceptor dysfunction and a vasoconstriction effect of decreased endothelin and nitric acid (NO) levels due to vascular endothelial dysfunction. These changes increase blood pressure, which increases ventricular tone due to stress, leading to heart failure [22].

However, periodic hypoxia, characterized by regular and predictable episodes of low oxygen levels, can contribute to the development of ASCVD by inducing oxidative stress and endothelial dysfunction [52]. These hypoxic events promote inflammatory responses and lipid deposition in arterial walls, accelerating the progression of atherosclerosis. Therefore, controlled studies on periodic hypoxia can provide insights into its impact on ASCVD, highlighting the importance of consistent oxygenation for cardiovascular health [53].

Table 1 (Ref. [1, 2, 23, 24, 54, 55, 56, 57, 58, 59]) lists studies linking OSA to atherosclerosis in the carotid artery. OSA patients had higher atherosclerotic plaque levels and narrower lumen diameters [1, 23, 54, 60]. OSA increases proinflammatory plasma cytokines levels, such as interleukin (IL)-2, IL-1, tumor necrosis factor (TNF)-α, polymerase chain reaction (PCR), and interferon-alpha (IFN-α), endothelial-dependent blood channel widening, and adhesion molecule activity [2, 55, 56]. Inflammatory markers are associated with increased risk for atherosclerosis [1, 61, 62]. A severe OSA lowers blood oxygen and raises blood pressure [59].

**Table 1.** **Relationships between OSA syndrome and carotid artery atherosclerotic disease**.

SN	Authors	Year	REF*	PS	REL*	Comorbidities	Progression of biomarkers	Relationship between the manifestation of OSA and CaAD
1	Hui *et al*. [23]	2012	37	50	OSA with cIMT	NR	CPAP treatment reduces plaques	CPAP treatment is useful in OSA patients
2	Nadeem *et al*. [24]	2013	38	1415	OSA with cIMT	HTN, DM	Increase in carotid diameter and carotid plaques	Increased plaque levels is symptomatic of an atherosclerotic process
3	Ciccone *et al*. [55]	2014	56	80	OSA with cIMT	HTN, pulmonary	Increased levels of plaques, CRP, IL-6, TNF, and PTX-3	The development of atherosclerosis may be influenced by inflammatory markers
4	Zhou *et al*. [57]	2017	58	18	OSA with carotid	HTN	Increase in atherosclerotic plaques	Independent risk factor for ASCVD
5	Song *et al*. [54]	2020	62	95	OSA with cIMT	NR	Increase in atherosclerotic plaques	Reduced plaque and carotid arterial elasticity
6	Bandi *et al*. [58]	2021	47	NR	OSA with HF	NR	Causes arrhythmogenicity and results in HF	Leads to atherosclerosis
7	Smith *et al*. [2]	2021	36	96	OSA with carotid	HTN	Elevation in important proinflammatory cytokines	The population’s inflammatory environment is a risk factor for childhood atherosclerosis
8	Suzuki *et al*. [56]	2022	31	07	OSA with carotid	HTN, DM	REM and OSA both are linked to metabolic and cardiovascular complications	Arterial stiffness and REM are increased
9	Gunnarsson *et al*. [1]	2014	28	790	OSA with carotid	HTN	Increase in carotid plaques	Increased future ASCVD and stroke risks
10	Firincioglulari *et al*. [59]	2022	23	190	OSA with carotid	NR	Increase in carotid artery calcification	More calcification leads to increased stroke risk

REF*, references in the respective articles; PS, patient size; REL*, relationship; OSA, obstructive sleep apnea; HTN, hypertension; cIMT, carotid intima-media thickness; DM, diabetes mellitus; HF, heart failure; CRP, C-reactive protein; NR, not reported; ASCVD, atherosclerotic cardiovascular disease; REM, rapid eye movement; CPAP, continuous positive airway pressure; CaAD, carotid artery disease; SN, serial number; IL-6, Interleukin-6; TNF, tumor necrosis factor; PTX-3, pentraxin-3.

Table 2 (Ref. [6, 7, 9, 16, 17, 63, 64, 65, 66, 67]) shows the link between OSA and coronary atherosclerotic disease. Long ST intervals (flat, isoelectric section on the electrocardiograph (ECG) between the end of the S wave and the beginning of the T wave) are connected to atherosclerosis [16, 68]. OSA was related to higher inflammatory activity in this CAD sample [63]. Moreover, left ventricle (LV) pressure and diastolic dysfunction result in left atrial hypertrophy [64, 69]. Periodic hypoxia can cause sympathetic activation, decreased parasympathetic tone, and inflammation [65, 70]. Studies have shown that obesity, hypertension, and diabetes are associated with OSA [17, 64, 66].

**Table 2.** **Relationship between OSA syndrome and coronary atherosclerotic artery disease**.

SN	Authors	Year	REF*	PS	REL*	Comorbidities	Progression of biomarkers	Relationship between the manifestation of OSA and CAD
1	Colish *et al*. [9]	2012	40	47	OSA with CAD	HTN	Risk of ASCVD	CPAP treatment is useful in OSA
2	Tan *et al*. [63]	2014	36	93	OSA with CAD	Obesity, HTN, DM	SA patients have increased atheroma volumes	Chronic OSA is an important potential risk factor for coronary atherosclerosis
3	Miller *et al*. [64]	2015	74	NR	OSA with CAD	Obesity, HTN	Risk of intermittent nocturnal hypoxemia	Atrial fibrillation
4	Valo *et al*. [16]	2015	39	80	OSA with CAD	NR	ST segment depression	Myocardial necrosis
5	Singh *et al*. [65]	2022	28	100	OSA with CAD	Obesity	Young patients with angiography show OSA serve symptoms	Correlation between obesity and OSA
6	Lu *et al*. [17]	2021	105	82	OSA with CAD	Obesity, HTN	Ventricular malfunction and remodeling	Coronary atherosclerosis
7	Tang *et al*. [67]	2021	28	158	OSA with CAD	HTN	Risk of ASCVD	Pulmonary hypertension
8	Liu *et al*. [66]	2022	44	255	OSA with CAD	Obesity, HTN	Patients in China with CAD have a significantly higher incidence of OSA	OSA is a risk factor for ASCVD
9	Wang *et al*. [6]	2023	45	1927	OSA with CAD	Hypertension	Ischemia-driven unstable angina	Acute coronary syndrome
10	Wojeck *et al*. [7]	2023	10	8246	OSA with CAD	NR	Ertugliflozin to reduce the effect of OSA	Sodium-glucose transporter 2 inhibitors are beneficial in OSA

REF*, references in the respective articles; PS, patient size; OSA, obstructive sleep apnea; DM, diabetes mellitus; HTN, hypertension; NR, not reported; ASCVD, atherosclerotic cardiovascular disease; CAD, coronary artery disease; CPAP, continuous positive airway pressure; SN, serial number; REL*: relationship; SA, sleep apnea.

## 4. Deep Learning for OSA Detection and ASCVD Risk Stratification

ML and DL have become more prominent in medical imaging [71, 72, 73]. The fundamentals of DL are based on deep neural networks (DNNs), a subgroup of DL that functions similarly to a human brain [74]. Recently, studies have used AI employing OSA detection in the HRV framework [75, 76, 77, 78] and ASCVD diagnosis and prognosis [28, 79, 80]. While DL is becoming popular for higher accuracy in segmentation and classification, hyperparameters need to be optimized during the training paradigm [31]. This requires several epochs, including optimal learning rate, batch size, batch normalization, and dropout layers, to avoid overfitting and achieve generalization without memorization [81]. To accomplish the foremost DL architecture, one must use multiple diagnostic sources with many distinct data sets in a big data framework [82]. However, comorbidities in patients alongside ASCVD and OSA influence non-linear dynamics between gold standard and covariates [83].

### 4.1 Deep Learning for OSA Detection 

**Fig. 3.**
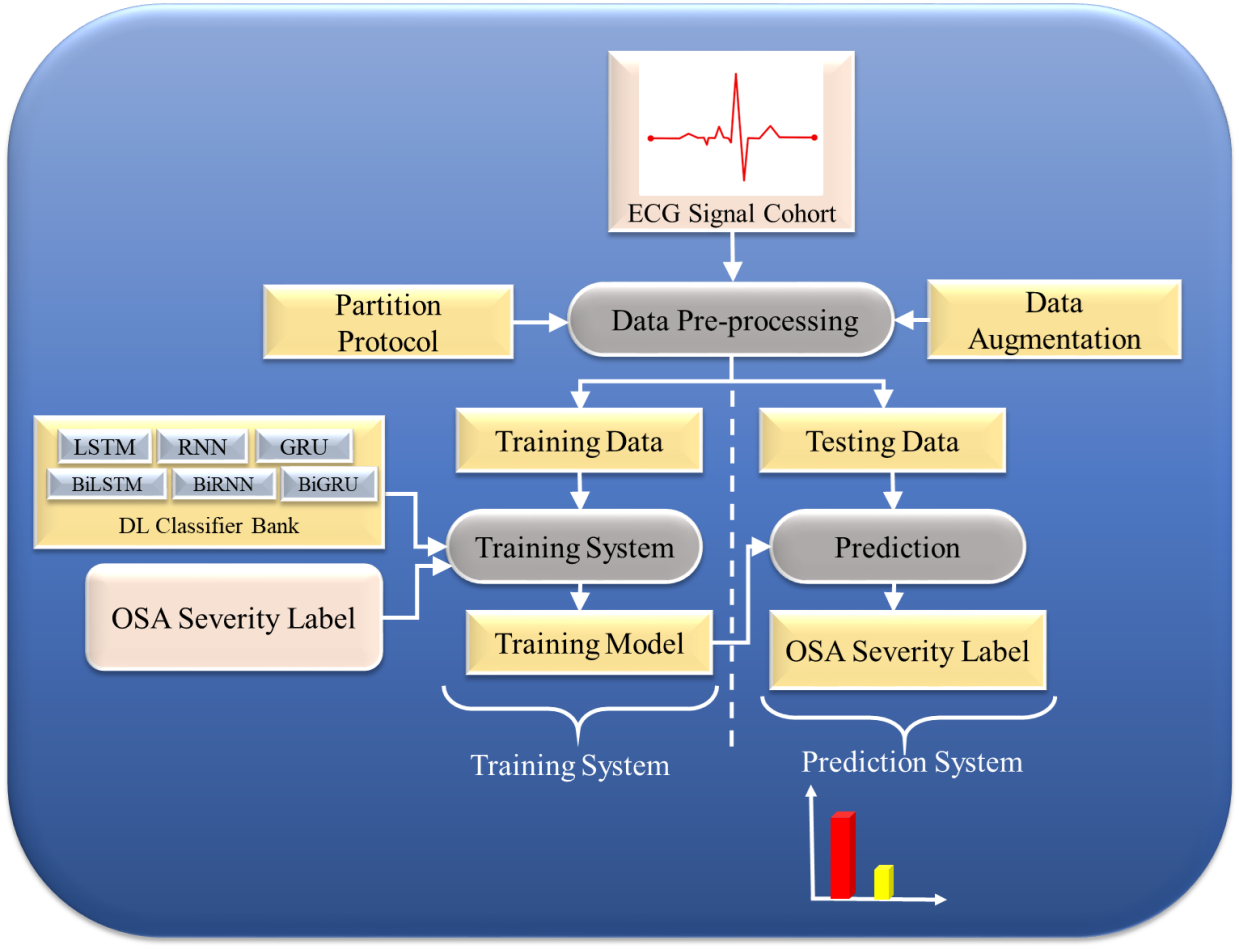
**Deep learning (DL)-based model for OSA prediction**. OSA, obstructive sleep apnea; ECG, electrocardiogram; LSTM, long short-term memory; RNN, recurrent neural network; GRU, gated recurrent unit; BiGRU, bidirectional gated recurrent unit; BiLSTM, bidirectional long short-term memory; BiRNN, bidirectional recurrent neural network.

The impulses from an ECG serve as the standard physiological measurement [84]. Interestingly, there is a strong connection between breathing disorders and ECG abnormality [85]. Hence, variation in the ECG can be a strong predictor of OSA [86]. Moreover, the processing and optimization of ECG signals are cost-effective [87]. DL algorithms train the model to extract features from polysomnography (PSG) signals automatically [84]. Overnight PSG measures numerous sleep parameters, while PSG recordings should be segmented into 30-second epochs to score sleep [88]. The training set trains the models, the validation set fine-tunes the models, and the testing set evaluates the performance. Subsequently, the data extracted from PSG determine the conclusion related to OSA severity. When large amounts of high-dimensional PSG data are available, DL models regularize the data and perform more accurate predictions than ML models [89, 90]. Singh and Talwekar [91] used hybrid deep learning (HDL) with CNN to predict OSA, achieving 80% accuracy. Locharla *et al*. [92] used DL with K-Nearest neighbors (KNN) and achieved 78% accuracy. Thus, DL systems for OSA prediction are powerful and reliable paradigms; however, work still needs to be performed to ensure the reliability and stability of the DL systems. Several studies that use AI for OSA prediction are shown in Appendix Table 4 (Refs. [20, 21, 33, 85, 89, 90, 93, 94, 95, 96, 97, 98]), Appendix Table 4. Fig. 3 shows a typical DL-based model for OSA prediction.

### 4.2 Deep Learning for ASCVD Risk Stratification 

**Fig. 4.**
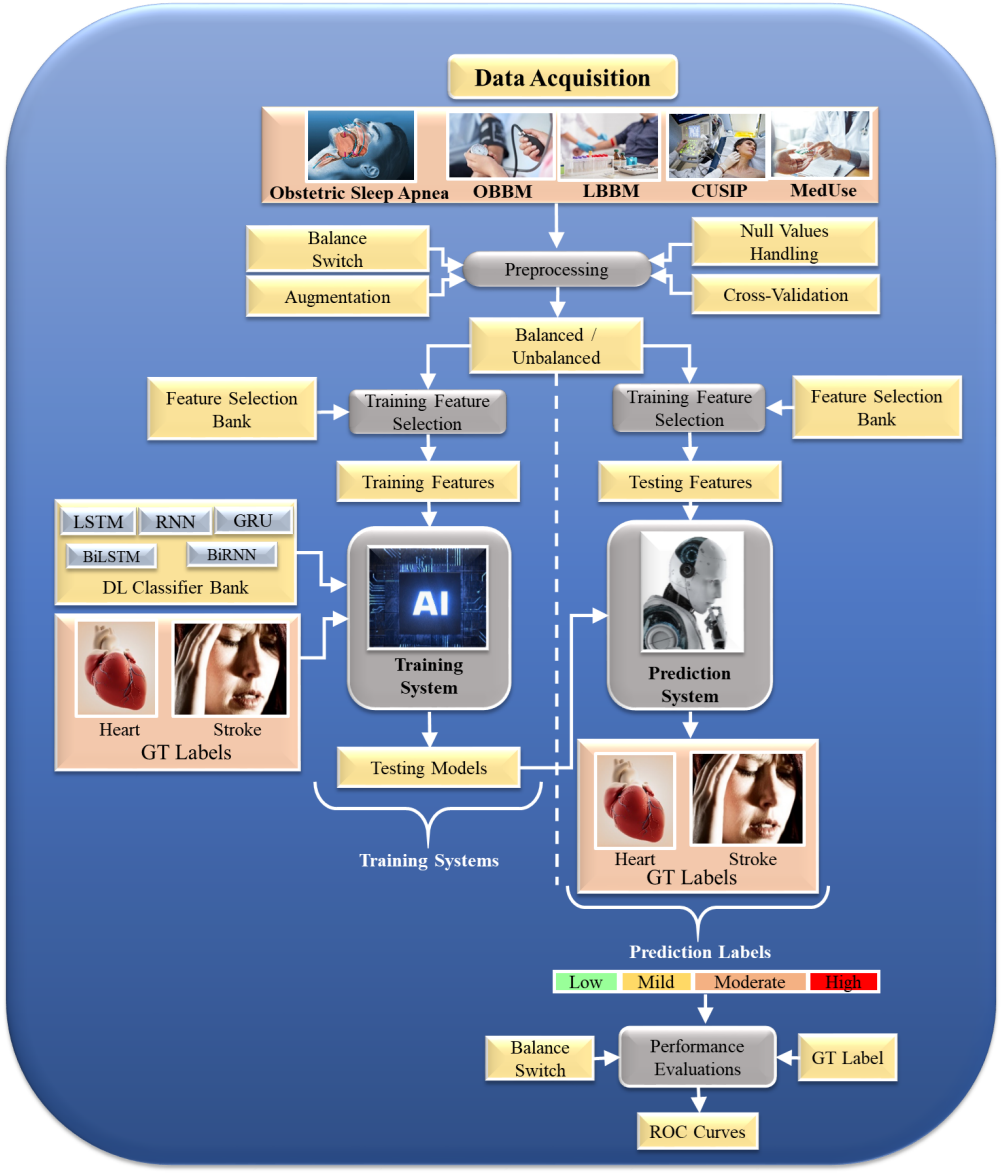
**OSA-based multiclass DL model to predict ASCVD/stroke severity**. OSA, obstructive sleep apnea; DL, deep learning; ASCVD, atherosclerotic cardiovascular disease; OBBM, office base bio markers; LBBM, lab base bio markers; CUSIP, committee on uniform securities identification procedures; MedUse, medication use; LSTM, long short-term memory; RNN, recurrent neural network; GRU, gated recurrent unit; BiLSTM, bidirectional long short-term memory; BiRNN, bidirectional recurrent neural network; GT, ground truth; AI, artificial intelligence; ROC, receiver operating characteristic.

DL is an effective strategy because it can use the underlying knowledge to create automated features and offers a better training paradigm that adjusts the non-linearity among both variables (covariates) and the gold standard. Fig. 4 depicts a typical DL system. This architecture comprises (a) a training design using composite risk factors such as OSA risk labels, office base bio markers (OBBMs), lab base bio markers (LBBMs), carotid ultrasound image phenotypes (CUSIPs), medication utilization (MedUSE), and (b) clinical risk labels representative of ground truth (GT), such as heart failure ASCVD and stroke [99]. This GT may indicate CAD, similar to a cardiac computed tomography (CT) score. Indeed, CT can be scored using DL. Suri *et al*. [100] have described CT-based grading. Intravascular ultrasound (IVUS) can also be used as a CT to represent CAD lesions [101, 102]. A non-linear training-based approach has been employed in heart disease risk stratification [103, 104].

**Fig. 5.**
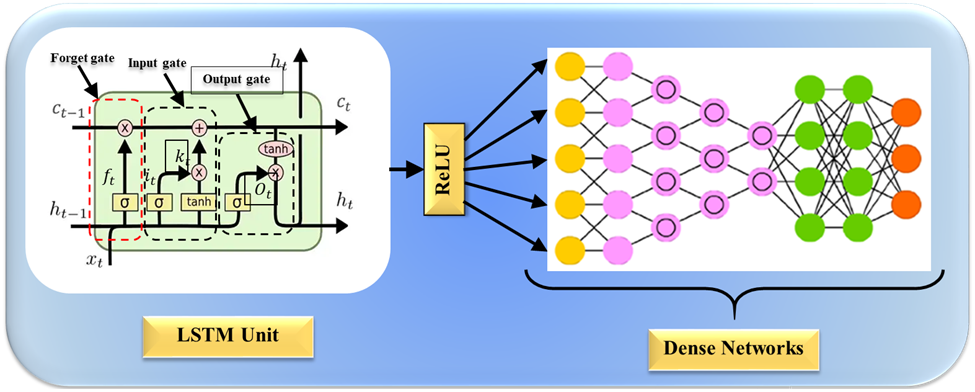
**Long short-term memory (LSTM) architecture for ASCVD risk stratification**. ASCVD, atherosclerotic cardiovascular disease; ReLU, rectified linear unit.

The recurrent neural network (RNN) and long short-term memory (LSTM) models can be used to evaluate sequential data, such as ECG, text [105], speech [106], and handwriting [107]. Further, these models contain a set of continuous data patterns. Previous RNNs could not learn long-term dependencies, resulting in a bridge problem connecting old and new data [108]. This problem further promoted the vanishing gradient problem, in which error signals vanished after backpropagation, leading to model failure [106]. LSTM has input, internal, and output gates: The input gate determines how much data will be forgotten; the internal gate determines the level of current state data; the output gate state is used to derive the next hidden state [109]. These models could memorize key data and understand long-term dependencies following the backpropagation of useful information. Fig. 5 depicts LSTM architecture.

### 4.3 Deep Learning for Plaque Wall Segmentation and CUSIP Measurements: A Surrogate Biomarker

**Fig. 6.**
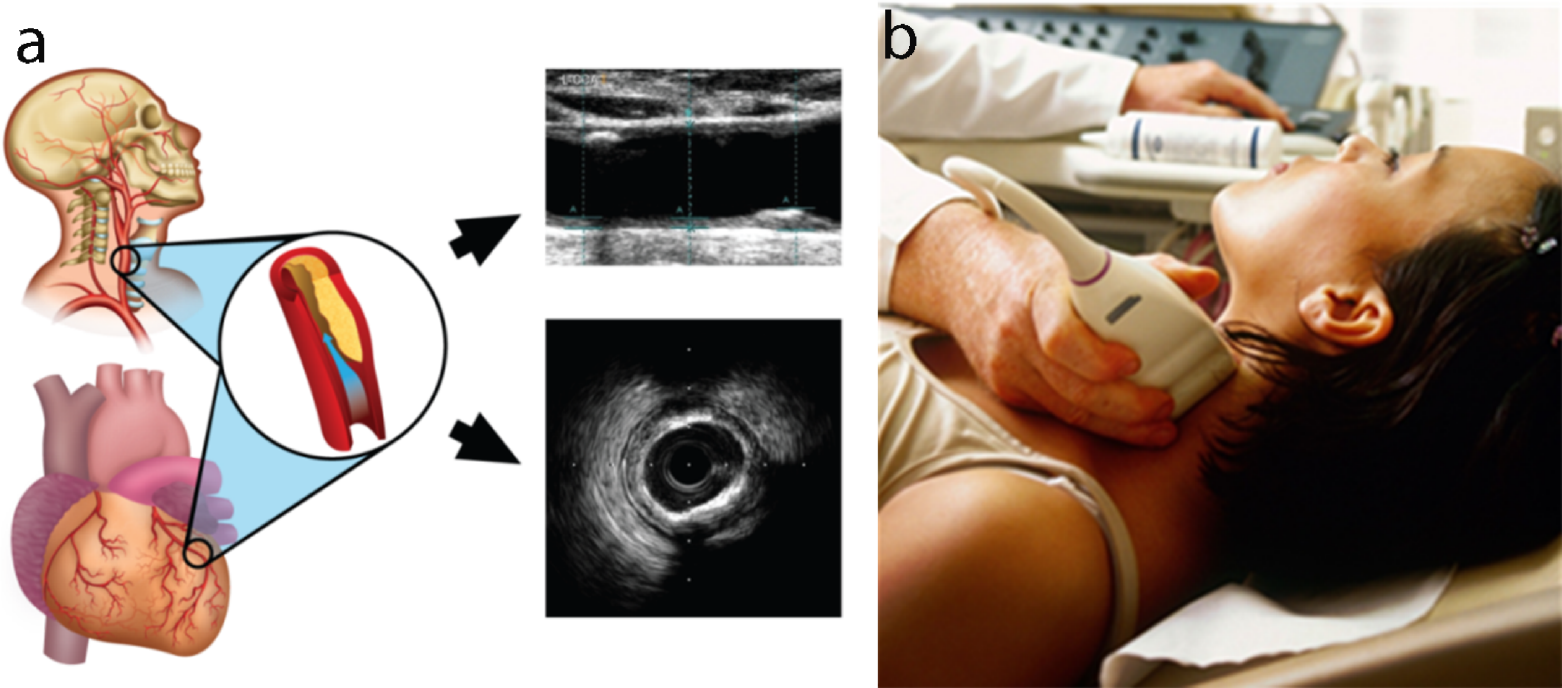
**The carotid artery examination using intravascular ultrasound (IVUS)**. (a) The carotid artery is a potential surrogate marker for the coronary artery, as shown by an IVUS-based vascular cross-sectional scan. (b) B-mode carotid longitudinal imaging system using linear ultrasound [112].

We have hypothesized that OSA leads to ASCVD disease via the morphological changes in the vascular network. CUSIPs refer to image-based carotid artery phenotypes [59, 110], and this training program is adaptable to non-linear adaptation [103, 104, 111]. Fig. 6 [112] represents the B-mode carotid ultrasound scan and its corresponding coronary atherosclerotic disease. The DL system can measure the atherosclerotic plaque area [113, 114]. The DL system computes the CUSIPs and helps to detect plaque aggregation in OSA patients [115]. Therefore, GT is required for DL-based ASCVD risk classification.

**Fig. 7.**
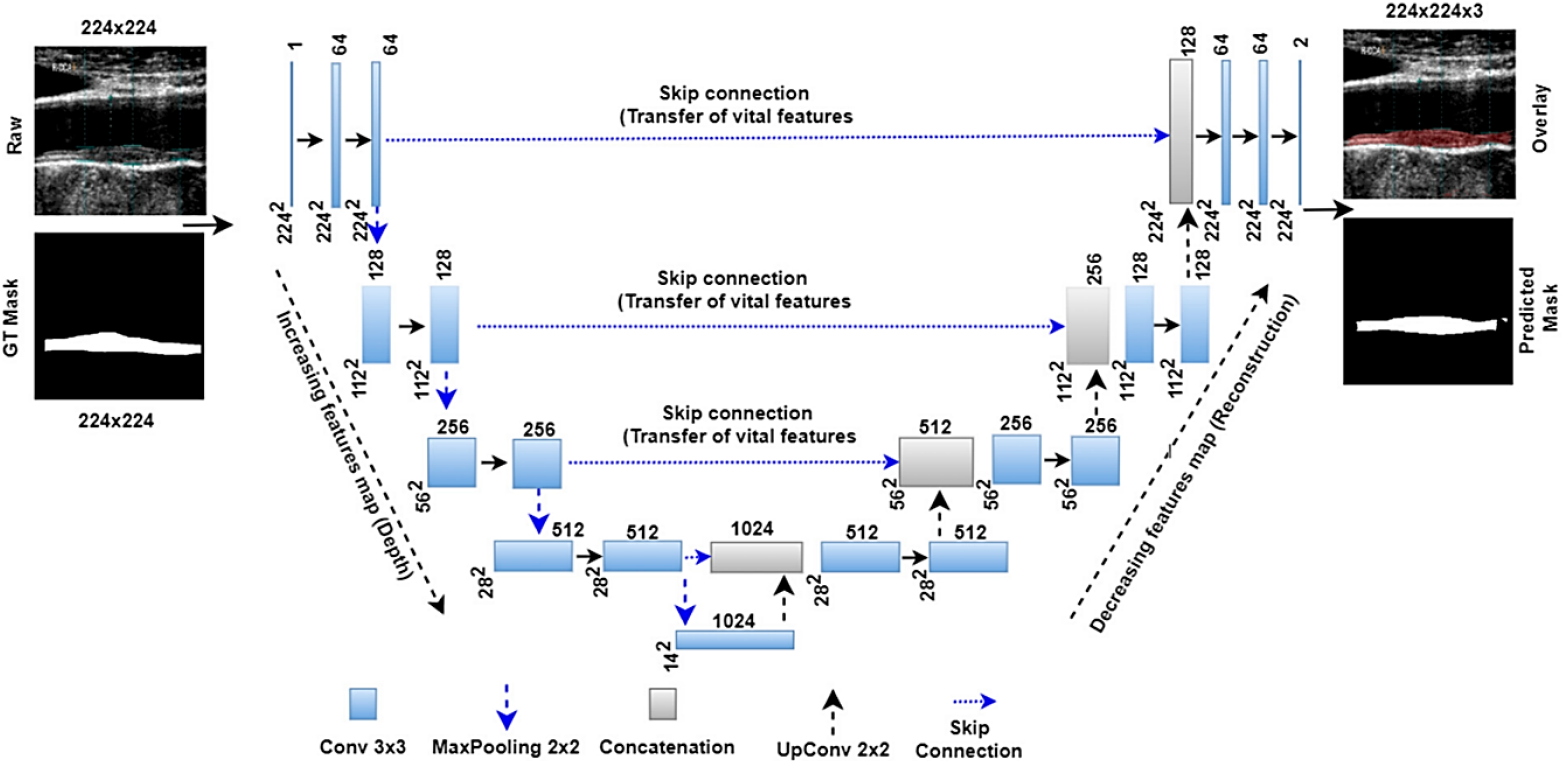
**UNet model for segmentation of the atherosclerotic plaque wall [116]**. UNet, U-Net (a type of convolutional neural network architecture for image segmentation); GT, ground truth.

Jain *et al*. [116] proposed a universal neural network (UNet) model to detect atherosclerotic plaques. The model uses four layers of deep learning (DL) and a pair of encoders and decoders. The model is shown in Fig. 7 (Ref. [116]). A sample is transmitted and received by the encoder. A two-dimensional-convolution rectified linear unit (ReLU) and MaxPooling may all be found in each UNet encoder layer. Each successive decoding stage employs up-convolution (two dimensions, 2D), depth-concatenation, and 2D convolution as part of image reconstruction.

## 5. Challenges and Recommendations in OSA Detection and ASCVD Risk Stratification

The DL system must address several key challenges to ensure the safety and efficacy of the medical devices used in ASCVD risk stratification for OSA patients: the comparison between carotid and retinal imaging, mitigating bias, ensuring explainability and trust in AI, optimizing ergonomics and cost-effectiveness, model pruning, cloud-based deployment, and the integration of carotid artery Doppler examinations.

### 5.1 A Special Note on Carotid Imaging vs. Retinal Imaging as Biomarkers for CVD Risk

**Fig. 8.**
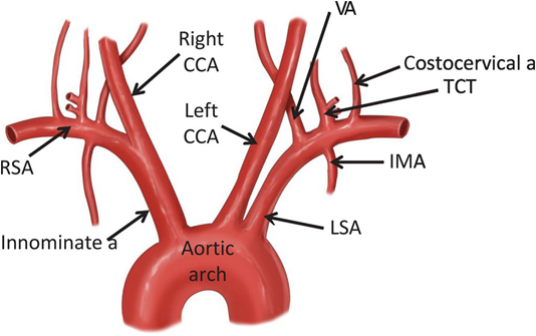
**The link between the coronary and carotid arteries [121]**. RSA, right subclavian artery; CCA, common carotid artery; VA, vertebral artery; TCT, total circulating tumour cell; MA, mammary artery; LSA, left subclavian artery.

The genetic makeup of the carotid artery: Previous studies have successfully established that the genetic makeup of the carotid artery is the same as the coronary artery [117]. Indeed, the coronary calcium volumes detected by IVUS are related to the automated carotid intima-media thickness (cIMT), which ensures the genetic nature of atherosclerosis disease. In another study, Araki *et al*. [118] used a machine learning classifier that used cIMT as the gold standard for risk stratification of coronary atherosclerosis disease, establishing the link between coronary plaques and subclinical atherosclerosis in the carotid artery, determining the genetic makeup of the atherosclerosis disease. The same pattern was established in other studies by Araki *et al*. [118], Banchhor *et al*. [101] and Narula *et al*. [119] . Lastly, it is easy to access the carotid artery from the innominate artery, i.e., where the innominate artery is connected to the aortic arch, which supplies blood to the brain and head [120], as the left common carotid artery branches off from the arch itself, and the right common carotid originates from the brachiocephalic trunk [101]. This anatomical connection is crucial in understanding the impact of aortic diseases, such as atherosclerosis, on cerebral blood flow and the potential risk of stroke, thereby highlighting the interdependency between the aorta and carotid arteries in vascular health shown in Fig. 8 [121]. A similar relationship exists between the coronary artery originating from the aortic arch, which supplies blood to the heart. The base of the aorta corresponds to the left and right coronary arteries (LCAs and RCAs) that supply blood to the heart, meaning the LCAs and RCAs are the first branches of the aorta. Thus, the two sets of arteries, coronary and carotid, originate from the aorta with the same genetic makeup [121, 122]. Since the genetic makeup of carotid and coronary arteries is similar, this study used the carotid arteries as a surrogate biomarker for coronary artery disease [101, 123].

A recent trend has emerged that uses the carotid artery as a biomarker instead of retinal scans to evaluate CVD risk stratification; this small note explains why the carotid artery is preferred over retinal scans. While this comparison is outside the scope of this review, we discuss the benefits the carotid artery offers. (i) The instrumentation for carotid artery imaging is ergonomic and user-friendly, meaning the sonographer, cardiologist, or radiologist can all perform carotid imaging without much training. The common carotid artery is easy to image using a linear transducer in an arterial cross-section mode and longitudinal modes [124, 125]. (ii) The economics of using carotid imaging over retinal imaging are favorable since the cost for carotid artery imaging is lower (economic) than retinal imaging devices. Meanwhile, the device used for retinal imaging is mentioned in the nutrition paper (diabetic retinopathy). Even though a normal camera can be used, the image quality is poor, meaning a stronger retinal imaging device is required, which is costly [126]. Recently, it was shown that when combined with AI, carotid imaging is very economical; however, surgeries can be prevented using carotid imaging [127]. (iii) Simplicity of processing images from carotid artery imaging: The image processing applied to carotid ultrasound is much simpler. This is because the lumen diameter is 10 mm, and the outer wall diameter is 12 mm [73, 101, 117, 128, 129]; meanwhile, the media-adventitial borders are 12 mm apart. Thus, the plaque burden is the difference between the outer and inner borders. (iv) East identification of media adventitia (MA) borders: The MA borders are easy to determine as there is a clear transition from the media region to the adventitia region, which indicates the MA borders of the carotid artery. Similarly, the lumen-intima (LI) borders are those between the lumen region (black region) and the media region (brighter region). Thus, the LI borders can also be easily estimated. The region between the LI and MA borders is the IMT region and represents the surrogate biomarker for coronary artery disease [130]. (v) Video imaging of the blood flow: Video imaging can also be taken when employing carotid artery imaging, which allows for stiffness computation, a very informative analysis for symptomatic vs. asymptomatic computations [102, 131, 132]. (vi) Deep learning paradigm: the feature extraction during the DL that represents closer to the coronary artery disease is the carotid artery.

This DL will likely provide a stronger design for CVD risk prediction, as proven before using a ML system design. Since the plaque burden in the carotid artery is a stronger biomarker for coronary heart disease (CHD), then there is a stronger chance that DL is more effective in carotid artery imaging (CAI) compared to retinal artery imaging (RAI). Carotid atherosclerosis plays a substantial role in cardiovascular morbidity and mortality. Given the multifaceted impact of carotid atherosclerosis, there has been increasing interest in harnessing AI and radiomics as complementary tools for the quantitative analysis of medical imaging data. This integrated approach holds promise in refining medical imaging data analysis and optimizing the utilization of radiologists’ expertise since AI allows radiologists to focus on more pertinent responsibilities by automating time-consuming tasks. Simultaneously, the capacity of AI in radiomics to extract nuanced patterns from raw data enhances the exploration of carotid atherosclerosis, advancing efforts in terms of (1) early detection and diagnosis, (2) risk stratification and predictive modeling, (3) improving workflow efficiency, and (4) contributing to advancements in research. This review provides an overview of general concepts related to radiomics and AI and their application in carotid-vulnerable plaques. It also offers insights into various research studies on this topic across different imaging techniques [133].

### 5.2 The Role of Bias in DL System Designs

Previous computer-aided diagnosis techniques lacked bias evaluations [83]; however, the importance of evaluating bias in AI models has increased significantly [134, 135]. Bias prevention can be handled using large sample sizes, proper clinical testing, introducing comorbidities, using big data configurations, unseen data analysis, and scientific validation of the training model design [100]. Identifying the AI risk of bias (RoB) [125, 136] and appropriately modifying diagnoses and treatments are key steps in patient risk stratification. Imbalanced data, common in medical datasets, can skew model performance towards over-represented classes. In the context of CVD prediction, we propose that strategies such as oversampling minority classes or undersampling majority classes can ensure the model learns equally from all categories of data. Synthetic data generation techniques such as the Synthetic Minority Oversampling Technique (SMOTE) or Adaptive Synthetic Oversampling Technique (ADASYN) [137] can also be applied in future implementations to balance class distributions without losing information [138]. Using diverse, multi-modal datasets that capture a wide range of patient demographics, symptoms, and conditions helps ensure that the model can generalize across various populations and is less likely to overfit specific groups. Furthermore, using large-scale datasets from different sources also reduces the risk of introducing bias from a single source [139].

### 5.3 Enhancing Model Explainability and Trust in AI

Understanding AI’s “black box” is one of the most critical challenges in its adoption. Moreover, providing clear explanations of AI model outcomes helps healthcare practitioners interpret and trust these results. Indeed, using tools such as Local Interpretable Model-agnostic Explanations (LIME) and SHapley Additive exPlanations (SHAP), which offer explainability for AI predictions, provides physicians with greater confidence in their models [73, 140]. Similarly, visualization techniques such as Gradient-weighted Class Activation Mapping (GradCAM), GradCAM+, and GradCAM++ can highlight lesions in carotid scans, aiding in interpretation [141]. These methods, which make the technology more transparent and user-friendly, can enhance the acceptance of AI in healthcare.

Explainability also makes AI systems more adaptable and cost-effective [142]. GradCAM and its variants are particularly useful for convolutional neural network (CNN)-based models such as carotid artery and retinal scans, which are commonly used in medical image analysis. These methods produce visual explanations by highlighting the areas in an image that contribute most to a prediction, thereby allowing clinicians to observe the specific features, such as artery blockages or other abnormalities, that influenced the model’s decision. This provides clinicians with a greater ability to validate or challenge AI-generated results [143, 144, 145].

LIME and SHAP provide valuable interpretability for non-image data, such as clinical, demographic, or symptom information. LIME creates local interpretable models by perturbing input features and observing changes in the output, helping clinicians understand how specific factors—such as apnea-hypopnea index (AHI) or oxygen desaturation levels in OSA patients—impact CVD or stroke risk predictions. SHAP complements this by providing a global interpretation, assigning consistent and additive importance values to each feature, and ensuring clinicians can identify the most critical factors in a patient’s risk profile [102]. Together, these tools improve the trustworthiness and clinical utility of AI models in healthcare.

Another way to improve trust is by integrating explainability tools such as GradCAM, LIME, and SHAP into clinical decision support systems. By providing real-time, transparent insights into the mechanisms through which models reach their decisions, clinicians can validate AI recommendations alongside their expertise, fostering a collaborative decision-making process [146]. Layer-wise Relevance Propagation (LRP) can further break down model predictions and assign relevance scores to individual neurons or pixels, especially in medical imaging models. This pixel-level breakdown explains how each input feature (such as a pixel in a medical scan) contributed to the final prediction, ensuring that decisions are not solely based on superficial or misleading information.

Finally, improving trust in DL models requires extensive testing and validation on diverse and representative datasets. Continuous evaluation of the performance of the models across different demographic groups, medical conditions, and geographic regions is essential to ensure the models perform robustly in various clinical settings. Future work should address potential biases during validation and improve the generalization ability of the models through fine-tuning and retraining.

### 5.4 The Role of Pruning-based DL Systems

As the Internet and cloud-based systems evolve, edge devices are gaining importance. Indeed, edge devices are particularly powerful when applying trained AI models for future predictions or disease risk stratifications. However, large data models are not deployable on edge devices, meaning compressed models are needed. Genetic algorithms (GA), particle swarm optimization (PSO), differential evolution (DE), and wolf optimization (WO) can be used to prune image-based DL models. Moreover, a fully connected network (FCN) or segmentation network (SegNet) can be used to compress the models [147].

### 5.5 Cloud-based Workflow in DL Models for ASCVD Risk Stratification for OSA Patients

**Fig. 9.**
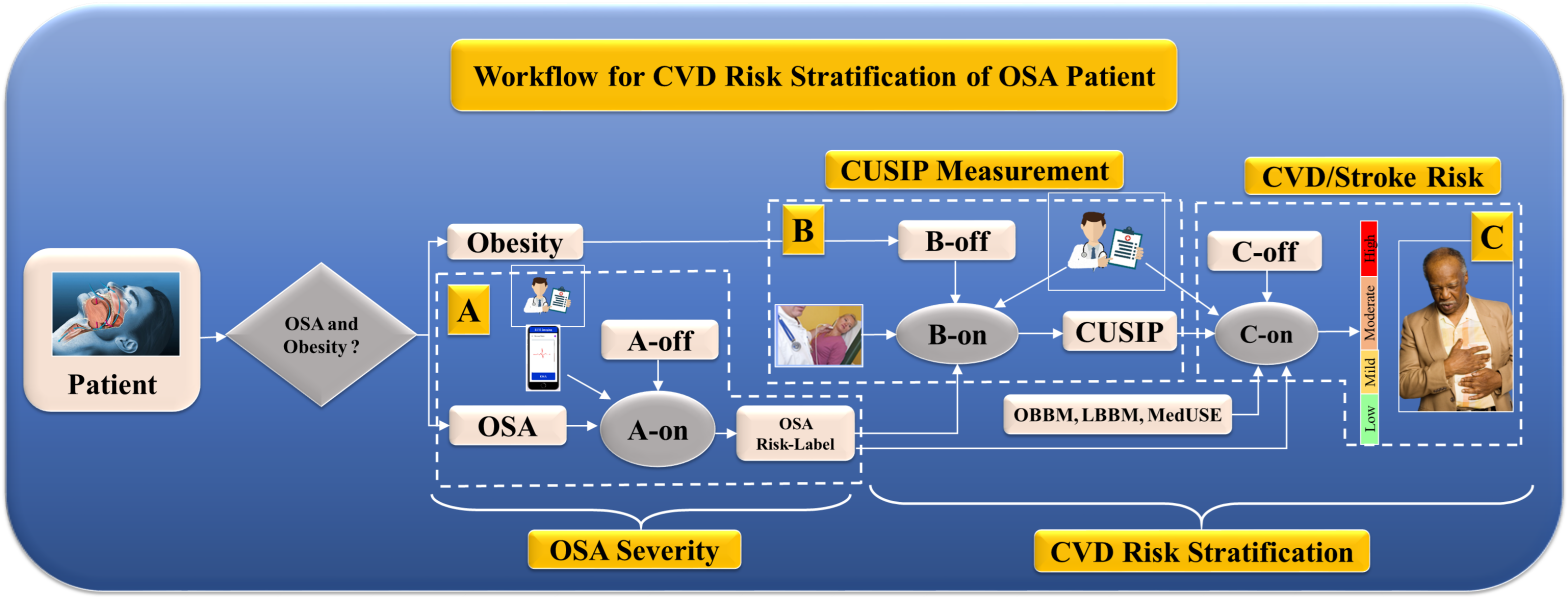
**The architecture of ASCVD screening for patients with OSA and obesity**. OSA, obstructive sleep apnea; CUISP, carotid ultrasound image phenotype; A-off, offline DL-based OSA training model; A-on, online DL-based OSA severity system; B-off, offline DL-based carotid training model; B-on, online DL-based carotid wall segmentation and quantification system; C-off, offline training-based ASCVD risk model; C-on, online DL-based ASCVD risk assessment; ASCVD, atherosclerotic cardiovascular disease; CVD, cardiovascular disease; DL, deep learning; OBBM, office base bio markers; LBBM, lab base bio markers; MedUSE, medication utilization; A, section A is OSA severity detection; B, section B is CUSIP measurement; C, section C is CVD/Stroke measurement.

As the Internet has become more technologically advanced, cloud-based technologies have evolved. Subsequently, we anticipate using cloud-based DL models to process OSA and ASCVD risk stratification [31, 148]. Fig. 9 depicts the role of DL in OSA detection and ASCVD risk stratification via a pipeline that contains A, B, and C cascaded systems. System A is used for OSA severity prediction on the test patient via A-on, given the A-off EEG-based OSA-trained model. System B is used for CUSIP segmentation via a DL-based UNet system, a B-on system, and a B-offline trained model. Finally, system C applies a DL-based ASCVD risk stratification using C-on via a C-off trained ASCVD model. The C-off LSTM-trained model uses biomarkers such as office base bio markers (OBBM), lab base bio markers (LBBM), CUSIP, MedUSE, OSA risk, and the ASCVD gold standard. The overall system uses smart-based OSA detection by analyzing real-time ECG signals during sleep, while system C uses cloud-based ASCVD risk stratification. Thus, the overall system is cost-effective.

### 5.6 Impact of Radiologist Experience on the Accuracy of Carotid Artery Doppler Examinations

An essential factor influencing the accuracy of carotid artery scanning is the experience level of the radiologists performing these procedures [149]. Experienced radiologists, often with several years of specialized training, can more precisely and efficiently image the common carotid artery, carotid bulb, and internal carotid artery [150]. Moreover, the expertise of the radiologist allows for minute pathological changes to be identified that may indicate atherosclerosis [151]. Comparatively, junior radiologists, who may still be refining their skills, could require more time to perform these examinations and additional training, particularly for imaging the more complex segments [152]. This difference in proficiency could potentially impact the diagnostic outcomes and should be considered when interpreting the results of studies involving carotid artery Doppler examinations [153]. Senior radiologists typically supervise procedures performed by junior radiologists to ensure consistency and accuracy in the imaging results.

### 5.7 Recommendations

ASCVD risk stratification in OSA patients has several recommendations: (i) Three hypotheses: (a) OSA leads to atherosclerosis formation promoting ASCVD, (b) DL systems are well suited for complex non-linear behavior undergoing morphological changes, and (c) combining OSA risk as a covariate with cardiovascular risk factors can improve ASCVD risk stratification; and recommendations are: (i) evaluation and validation: DL systems must undergo a clinical evaluation and scientific validation for a robust OSA detection and ASCVD risk stratification; (ii) hyperparametrization: DL systems must be hyperparameterized in both phases: (a) OSA detection and (b) ASCVD risk stratification; (iii) bias: bias in DL models can be best reduced by balancing the risk classes (control, low-risk, and high-risk); (iv) edge devices: DL systems can be ported to edge devices if these systems are pruned or compressed; (v) carotid surrogate imaging: DL systems based on surrogate carotid imaging can be low-cost without reducing ASCVD risk stratification accuracy.

## 6. Discussion 

### 6.1 Principal Findings

This study emphasizes four major objectives:

(i) Establishment of the link between OSA and heart failure;

(ii) Role of DL for CVD risk stratification in OSA patients;

(iii) Role of surrogate biomarkers for CVD risk;

(iv) Fusion of OSA risk factors with other risk factors for composite CVD risk.

(i) Link between OSA and heart failure: OSA is intricately linked to numerous cardiovascular complications, including heart failure. The relationship between OSA, ASCVD, and stroke is complex and involves various pathogenic mechanisms. Studies have shown that OSA contributes to conditions such as intermittent hypoxia, hypercapnia, oxidative stress, systemic inflammation, and endothelial dysfunction, all of which are critical in the development of ASCVD and stroke. OSA-induced intermittent hypoxia leads to oxidative stress and systemic inflammation, which promote the development of atherosclerotic plaques. These plaques cause cardiovascular complications by inducing endothelial dysfunction and increasing the deposition of LDLs in arterial walls [44, 154]. Additionally, research has demonstrated that OSA patients have higher levels of inflammatory markers and greater carotid artery atherosclerosis compared to non-OSA individuals [63]. OSA has also been identified as an independent risk factor for stroke, with mechanisms such as hemodynamic instability, increased sympathetic activity, and impaired cerebral autoregulation playing significant roles [7].

(ii) Role of DL for carotid imaging: DL techniques have shown remarkable capabilities in medical imaging analyses, providing accurate and efficient predictions for various health conditions [155, 156, 157]. Thus, applying DL to carotid imaging in OSA patients offers a promising approach to stratifying ASCVD and stroke risks. DL algorithms, particularly CNN, have effectively identified and quantified atherosclerotic plaques in carotid imaging, offering detailed insights into the severity and progression of ASCVD [81, 147, 158, 159]. DL models trained on large datasets of carotid images can accurately predict cardiovascular events by analyzing plaque characteristics such as size, composition, and morphology [81, 141, 160].

(iii) Role of surrogate biomarker for CVD risk: These predictions are crucial for risk stratification in OSA patients, who are at higher risk for ASCVD and stroke. Integrating DL-based carotid imaging analysis with traditional cardiovascular risk factors enhances the precision of risk prediction models, providing more personalized and effective management plans for OSA patients [125, 161, 162].

(iv) Fusion of OSA risk factors with other risk factors for composite CVD risk: Incorporating OSA risk into cardiovascular risk models can significantly improve the accuracy of predicting adverse cardiovascular outcomes, similar to erectile dysfunction [160] or Parkinson’s disease [163, 164]. Traditional risk models for CVD and stroke typically consider factors such as age, sex, hypertension, diabetes, cholesterol levels, and smoking status [165, 166]. Therefore, adding OSA risk to these models acknowledges the substantial impact of sleep-disordered breathing on cardiovascular health. Studies have shown that OSA independently predicts cardiovascular events and its inclusion in risk models can improve the stratification of patients into different risk categories, allowing for more targeted and effective interventions [167, 168].

Validating these enhanced models through clinical trials and longitudinal studies can provide robust evidence for the utility of including OSA risk in cardiovascular risk stratification, ultimately leading to better outcomes for patients with OSA [64].

### 6.2 Benchmarking

Table 3 (Ref. [169, 170, 171, 172, 173, 174, 175, 176, 177]) provides a comprehensive benchmarking analysis of various studies that predict ASCVD risk in OSA patients using AI technologies. Table 3 includes 15 attributes: serial number, studies, year, references, comorbidities, body mass index (BMI), ethnicity, ECG, waist circumference, polysomnography (PSG), electromyography, oxygen saturation, AI type, Food and Drug Administration (FDA) discussion, clinical setting, and risk of bias. Key observations from Table 3 indicate that only two studies, Cao *et al*. [169], and Brennan and Kirby [170], specifically predicted ASCVD in OSA patients using DL. The remaining studies focused on predicting OSA alone. Among these studies, seven had hypertension as a comorbidity, nine utilized DL technologies, and two employed ML methods. Mostafa *et al*. [171] used DL with a decision tree (DT) classifier to predict OSA in hypertensive patients, achieving an accuracy of 85%. Munjral *et al*. [26] also used DL but with a random forest (RF) classifier, achieving 87% accuracy. Cao *et al*. [169] predicted both ASCVD and hypertension using DL with a convolutional neural network (CNN), reaching 90% accuracy. Cao *et al*. [169] combined DM with ASCVD and hypertension in their predictions using DL with RF, achieving 88% accuracy. Hong *et al*. [172] included DM and hypertension and used DL with KNN, achieving 82% accuracy. Qian *et al*. [173] used ML with a DT classifier to predict OSA in hypertensive patients and achieved 84% accuracy. Brennan and Kirby [170] achieved 89% accuracy using DL with RF, covering multiple comorbidities, including DM, ASCVD, and hypertension. Ferreira-Santos *et al*. [174] used ML with eXtreme gradient boosting (XGB) to predict OSA in patients with DM and hypertension, achieving 86% accuracy. Singh and Talwekar [91] used HDL with CNN for OSA predictions, achieving 80% accuracy. Locharla *et al*. [92] used DL with KNN, achieving 78% accuracy. The most recent study by V. Kumari *et al*. [175] used DL with CNN to predict ASCVD, achieving the highest accuracy of 91%. This presented study aimed to predict both OSA and ASCVD, including comprehensive physiological parameters, but lacked the risk of bias discussion.

**Table 3.** **Benchmarking data for ASCVD risk in OSA patients**.

K0	K1	K2	K3	K4	K5	K6	K7	K8	K9	K10	K11	K12	K13	K14	K15	K16	K17
1	Mostafa *et al*. [171]	2019	93	HTN	✓	✘	✘	✘	✓	✘	✘	DL	✓	✓	✘	DT	85%
2	Pépin *et al*. [176]	2020	85	OSA	✓	✘	✘	✘	✘	✘	✘	DL	✓	✓	✘	RF	87%
3	Loh *et al*. [177]	2020	106	ASCVD, HTN	✓	✘	✓	✘	✓	✓	✓	DL	✘	✓	✘	CNN	90%
4	Cao *et al*. [169]	2020	72	DM, ASCVD, HTN	✓	✘	✓	✘	✓	✓	✓	DL	✘	✘	✘	RF	88%
5	Hong *et al*. [172]	2020	231	DM, HTN	✘	✓	✓	✘	✓	✓	✓	DL	✘	-	✘	KNN	82%
6	Qian *et al*. [173]	2021	155	HTN	✘	✘	✓	✘	✓	✓	✓	ML	✘	✓	✘	DT	84%
7	Brennan *et al*. [170]	2022	63	DM, ASCVD, HTN	✓	✓	✓	✓	✓	✓	✓	DL	✓	✓	✘	RF	89%
8	Ferreira-Santos *et al*. [174]	2022	68	DM, HTN	✓	✘	✓	✓	✓	✓	✓	ML	✘	✓	✘	XGB	86%
9	V. Kumari *et al*. [175]	2023	275	ASCVD	✘	✘	✘	✘	✘	✓	✓	DL	✘	✘	✘	CNN	91%
10	Maindarkar *et al*. (proposed)	2024	144	OSA, ASCVD	✓	✓	✓	✓	✓	✓	✓	✓	✓	✓	✓	✘	✘

K0, serial number; K1, studies; K2, year; K3, references; K4, comorbidities; K5, body mass index; K6, ethnicity; K7, electrocardiograph; K8, waist circumference; K9, polysomnography; K10, electromyograph; K11, oxygen saturation; K12, AI type; K13, FDA discussion; K14, clinical setting; K15, risk of bias; K16, classifier; K17, accuracy of the model; DM, diabetes mellitus; ASCVD, cardiovascular disease; HTN, hypertension; DL, deep learning; ML, machine learning; OSA, obstructive sleep apnea; DT, decision tree; RF, random forest; CNN, convolutional neural network; KNN, K-Nearest neighbors; XGB, XGBoost (Extreme Gradient Boosting); ✓, included; ✘, exculded.

Notably, only six studies [169, 172, 173, 174, 175, 177] discussed FDA regulations, which are crucial for product design and clinical application. The risk of bias was not discussed in any study except the proposed one, highlighting a gap in addressing potential biases in the research methodologies.

### 6.3 Strengths, Weakness, and Extensions

The primary strength of this review lies in its introduction of ASCVD risk stratification, which was specifically tailored for patients with OSA. This review highlights the complex biological and morphological interactions between OSA and ASCVD, forming the basis of our first hypothesis. We propose a DL solution for the dual tasks of OSA detection and ASCVD risk prediction. Our system is a cascaded framework that integrates the computation of OSA risk labels, CUSIP segmentation, and ASCVD risk stratification using LSTM models.

Despite the simplicity of the proposed system, there are important considerations for its optimization. One key aspect is minimizing the risk of bias, which is critical for ensuring the reliability and validity of the DL models. Additionally, the system needs to be generalized to account for various comorbidities often coexisting with OSA, thereby improving its robustness and applicability across diverse patient populations. This necessitates rigorous testing and validation across different datasets and clinical scenarios.

To further enhance the performance and accuracy of the system, we suggest extending the design to incorporate ensemble-based DL systems. Ensemble methods, which combine multiple models to improve predictive performance, can help address the limitations of individual models and provide more reliable predictions. By leveraging ensemble techniques, the system can achieve higher accuracy in OSA detection and ASCVD risk stratification, ultimately leading to better clinical decision-making and patient outcomes.

## 7. Conclusions

This review presented three key hypotheses that form the foundation of its analysis. First, it explored the biological link between OSA and ASCVD, highlighting the complex interplay of factors contributing to both conditions. Second, it proposed that incorporating OSA risk into existing models could significantly improve the stratification of ASCVD risk, suggesting that understanding and quantifying OSA can provide valuable insights into cardiovascular health. Third, it examined the capability of DL to manage the intricate relationships involved due to its sophisticated layers and superior feature extraction methods.

A clear and detailed connection was established between OSA and atherosclerotic disease, particularly in critical areas such as the carotid arteries, coronary arteries, and aorta. This review highlighted the efficacy of DL models in detecting OSA, utilizing this detection as a critical biomarker. This biomarker was combined with other office-based and laboratory-based biomarkers, carotid ultrasound imaging phenotypes, and statin usage data to create a comprehensive ASCVD risk stratification model. This multi-faceted approach aimed to provide a more accurate and personalized risk assessment for patients with OSA.

This review also delved into several significant challenges that need to be addressed to enhance the application of AI in this context. These challenges include AI bias, which can skew results and reduce the reliability of predictions; AI explainability, which is crucial for gaining clinical trust and understanding the decision-making process of DL models; AI pruning, which involves reducing the complexity of models to make them more efficient without sacrificing accuracy. Additionally, this review proposed a cloud-based cascaded system designed to provide a personalized approach to ASCVD risk stratification, leveraging the scalability and accessibility of cloud computing to implement these advanced AI methods effectively.

## Abbreviations

ACC, American College of Cardiology; ARDS, acute respiratory distress syndrome; ASCVD, atherosclerotic cardiovascular disease; ANS, autonomic nervous system; AUC, area-under-the-curve; AI, artificial intelligence; BMI, body mass index; CAD, coronary artery disease; CAS, coronary artery syndrome; CHD, coronary heart disease; CT, computed tomography; CUSIP, carotid ultrasound image phenotype; CV, cross-validation; CVD, cardiovascular disease; CVE, cardiovascular events; CNN, convolution neural network; DL, deep learning; DM, diabetes mellitus; EEGS, event-equivalent gold standard; EMG, electromyography; EC, endothelial cell; GT, ground truth; HTN, hypertension; HDL, hybrid deep learning; ICAM, intercellular adhesion molecule; VCAM, vascular cell adhesion molecule; LBBM, lab base bio markers; MRI, magnetic resonance imaging; NR, not reported; NPV, negative predictive value; NB, naive bayes; NO, nitric oxide; nOH, neurogenic orthostatic hypotension; Non-ML, non-machine learning; OBBM, office base bio markers; OH, orthostatic hypotension; OxLDL, oxidation of low-density lipoprotein; OSA, obstructive sleep apnea; PE, performance evaluation; PPV, positive predictive value; PCA, principal component analysis; PRISMA, Preferred Reporting Items for Systematic Reviews and Meta-Analyses; PTC, plaque tissue characterization; RA, rheumatoid arthritis; PR, the period measured in milliseconds; RF, random forest; ROS, reactive oxides stress; RoB, risk of bias; ROC, receiver operating-characteristics; RNN, recurrent neural network; SCORE, systematic coronary risk evaluation; SMOTE, synthetic minority over-sampling technique; SVM, support vector machine; SAA, sleep apnea association; TPA, total plaque area; TC, tissue characterization; US, ultrasound.

## Appendix

See Appendix Table 4.

**Table 4.** **DL-based OSA studies use ECG alongside risk factors and GT as input modalities**.

SN	Studies	Year	REF	DS	MOD	IC	Risk factors							GT
							R1	R2	R3	R4	R5	R6	R7	
1	Anitha *et al*. [85]	2021	31	285	LBBM, OBBM	ECG	✘	✘	✓	✘	✓	✘	✘	OSA, non-OSA
2	Kim *et al*. [93]	2021	45	279	LBBM, OBBM	ECG	✓	✓	✓	✘	✘	✘	✘	OSA, non-OSA
3	Ma *et al*. [94]	2021	71	2277	LBBM, OBBM	ECG	✓	✓	✓	✓	✓	✓	✓	OSA, non-OSA
4	Panindre *et al*. [95]	2021	169	30	LBBM, OBBM	ECG	✓	✓	✓	✓	✘	✓	✓	OSA, non-OSA
5	Brink-Kjaer *et al*. [33]	2022	77	2500	LBBM, OBBM	ECG	✘	✘	✘	✘	✓	✘	✘	SA, life expectancy
6	Almutairi *et al*. [90]	2021	71	70	LBBM, OBBM	ECG	✓	✘	✓	✘	✘	✘	✘	OSA, non-OSA
7	Tsai *et al*. [96]	2022	69	10,391	LBBM, OBBM	ECG	✓	✘	✓	✓	✓	✘	✘	OSA, non-OSA
8	Ramesh *et al*. [89]	2021	80	1500	LBBM, OBBM	ECG	✓	✓	✓	-	✓	✘	✘	OSA, non-OSA
9	Gourishetti *et al*. [20]	2022	39	6814	LBBM, OBBM	ECG	✓	✓	✓	✓	✓	✓	✓	OSA, ASCVD
10	Samadi *et al*. [97]	2022	33	18	LBBM, OBBM	ECG	✓	✓	✓	✓	✓	✓	✓	OSA, non-OSA
11	Tasmi *et al*. [21]	2022	43	136	LBBM, OBBM	ECG	✓	✓	✓	✓	✓	✓	✓	SA, mortality
12	H. Liu *et al*. [98]	2023	36	70	LBBM, OBBM	ECG	✓	✓	✓	✘	✘	✓	✓	SA, mortality

REF, references in the respective articles; DS, data size; IC, input covariates; MOD, modality; LBBM, lab base bio markers; OBBM, office base bio markers; ECG, electrocardiograph; GT, ground truth; R1, body mass index; R2, hypertension; R3, electrocardiogram; R4, waist circumference; R5, polysomnography; R6, electromyography; R7, oxygen saturation; DL, deep learning; OSA, obstructive sleep apnea; SN, serial number; SA, sleep apnea; ASCVD, atherosclerotic cardiovascular disease.
